# Selections

**Published:** 1891-05

**Authors:** 


### Selections.

MARRIAGE AND CHRONIC GONORRHŒA.—Dr. G. E. Br wn, of New York, presents a valuable paper on this subject in the *International Journal of Surgery* for February. Concerning syphilis and marriage, he says, a great deal has been said, and he is surprised that even in recent works gonorrhœa, in its matrimonial relations, should receive so little attention. The opinion is widely held now that far more suffering and incurable disease in women can be attributed to gonorrhœal than to syphilitic infection.

It has not infrequently been my experience to be consulted by young men, a few weeks or months before contemplated marriage, with a history of one or more attacks of gonorrhœ a informer years, who believe themselves to be well; yet who, upon a careful examination, present the unmistakable signs of a chronic urethritis.

The only evidence of disease remaining in these cases may be, and frequently is, the presence in the urine of small, thread-like bodies, to which the name of *tripper faden* has been given by the German surgeons who first described them and demonstrated their importance. These minute shreds are composed of mucus, pus and epithelium, and represent the secretions which adhere to any granular patch or area of chronic inflammation remaining on the urethral mucous membrane.

Noeggerath concludes that a man who has once been the subject of a gonorrhœal urethritis never fully recovers; that the disease lingers in the glands and ducts emptying into the canal, and may at any time furnish a secretion which may infect those with whom he has sexual relations. He also states that nine-tenths of all women married to men who have had gonorrhœa, sooner or later become the subject of incurable and painful inflammatory disease of the uterus, tubes or ovaries; that this infection may take place rapidly, and manifest itself as an acute infection, or by means of a slow and unrecognized process to which he gives the name of "latent gonorrhœa." He further adds that 90 per cent. of all cases of sterility can be directly traced to gonorrhœa.

Ernest Finger states, regarding marriage, that it should be absolutely prohibited in all cases where the existence of a chronic urethritis is evidenced by the presence of the "morning drop" or *tripper faden* in the urine, until the following facts have been established:

1. That after from two to four weeks of daily observation, the secretions from the urethra are found to be *free from pus and made up wholly of epithelial cells.*

2. That no gonococci can be detected by the microscope, even after a purulent discharge has been established by the employment of irritating injections of corrosive sublimate or nitrate of silver.

3. That neither prostatitis nor stricture exists.

Dr. Brewer then recites a case in his own experience which presented the above matrimonial contraindications. Against his advice and warning the young man married. Consequently, in two weeks time he saw the young wife with a purulent urithritis and vulvitis, pus showing abundant colonies of gonococci. A severe cystitis followed, a large utero-vaginal abscess which was opened under ether; also a marked pyelitis which continued for months.

In reviewing the records of nearly one thousand cases of urethritis treated by me during the past five years I find that in six instances I was consulted regarding the propriety of marriage, under circumstances similar to those which existed in the case reported above. My rule had always been in such cases never to allow marriage until at least three months had elapsed since the cessation of all acute symptoms, and until repeated examinations of the secretions (including the *tripper faden*) had failed to show the presence of gonococci. In the six cases referred to these conditions were observed, and in no instance has the wife exhibited the slightest evidence of infection.

In conclusion, allow me to urge upon all interested in this subject the necessity of unusual care in examinations undertaken with a view to forming an opinion regarding the propriety of marriage in those who have been the subject of gonorrhœal urethritis. The safest method would be to follow the advice of Finger as quoted above. Certainly, no one should assume the responsibility of sanctioning a marriage, without at least imposing the conditions which it has been my custom to insist upon.

---

THE USELESSNESS OF SPLINTS IN FRACTURE OF THE LOWER END OF THE RADIUS.—In an article on this subject (*Medical News*) Dr. John B. Roberts, Philadelphia, maintains that the use of splints is not at all necessary in the treatment of Colles' fracture of the radius. The treatment of these fractures is exceedingly satisfactory ; the results obtained are usually good, both in rapidity of cure and perfect restoration of function.

The usual cause of the injury is forced extension of the radio-carpal joint, which produces a transverse disruption through the lower end of the radius from three-eighths to one-half an inch above the articular surface. The characteristic deformity is caused by the fracturing force driving the lower fragment upward and backward upon the shaft, or thrusting the shaft downward and under that fragment, so that it is caught or impacted upon the dorsal edge of the shaft-fragment. Occasionally there is a tendency to lateral or antero-posterior obliquity of the line of fracture, but this is rather uncommon. Sometimes comminution of the lower fragment takes place so that lines of fracture enter the radio-carpal joint. The ligaments and cartilages are sometimes extensively injured, and sometimes there occurs actual loss of substance by crushing and pulverizing of the bone tissue. These complications, except that of comminution, are quite rare.

Reduction of the fracture, the most important element in the treatment of the injury, is often ineffectually accomplished, or, indeed, not attempted. This is owing to ignorance rather than carelessness on the part of the attendant. When reduction is once thoroughly accomplished, displacement is not apt to recur, because the broad rough surfaces of bone are held together by their serrations, and because there are no muscular masses tending to displace the fragments.

The condition, it will be observed, is quite different from oblique fracture of the shaft of a bone, in which it is often difficult to maintain accurate apposition because of the muscular displacing forces. Hence if reduction, which is the essential in treatment, is properly performed, no splint is needed. On the other hand, if reduction is neglected, no splint will act as a substitute for it. If reduction has been properly accomplished, an improper splint may displace the lower fragment and cause recurrence of the deformity. Hence, abandonment of splints is usually the proper course to pursue, and probably the most judicious method of treatment to advocate and teach.

Comminuted fractures, of course, need more support than do non-comminuted ones ; but even here, the simple support of a bandage applied in a circular manner, or of strips of adhesive plaster wound around the wrist like a collar will usually be found sufficient.

At the present time I should be inclined in nearly all cases to treat the fracture without using any splint at all ; or, at most, I should employ only a thin strip of steel or zinc, or a couple of pieces of whalebone, six inches long, applied to the dorsum of the wrist, and held in place by strips of adhesive plaster. I am now convinced that a roller bandage or a strip of adhesive plaster applied to the wrist in a circular manner is all that is necessary, except in unusually complicated fractures. All ordinary forms of splints should, as a rule, be discarded as useless or dangerous.

The proper treatment of fracture of the lower end of the radius is *reduction.* Little else is required in the ordinary cases.

---

NEW METHODS OF TREATMENT IN TYPHOID FEVER.—Dujardin Beaumetz (*Bullet. Gen. de Theraput.*) studies prophylaxis of typhoid fever, and the use of cold baths, intestinal antiseptics, and diuresis in the treatment of the disease.

Prophylaxis has been more benefited than was treatment by the discovery of the bacillus of Eberth. Rigorous care of typhoid patients is insisted upon ; a strong solution of copper sulphate, twelve drachms to the quart, is recommended for washing sheets, etc. ; a weaker solution, three drachms to the quart, is used to disinfect the nurses hands, etc. For intestinal antisepsis salol is preferable to iodoform, naphthol, naphthaline, etc., being less harmful and more efficient. Thirty to sixty grains may be given in twenty-four hours, in combination with salicylate of bismuth, if the drug is indicated.

Hyperexia is not an essential part of the general condition of the disease. The case may be serious without it. Antipyretic drugs decrease urinary secretion, and retard the elimination of the poisons produced. The author finds that in sponging, enveloping in wet sheets and tepid baths, he has all the advantages of cold baths without their inconveniences. He treats cases symptomatically. Beginning with sponging, he gives tepid baths (86°-89° F.) ; if the temperature passes 104° F., one or two a day, lasting twenty to thirty minutes. Stimulating drinks are given to patients, if needed, while in the bath.

Quinine is regarded as the drug best able to met the general indications of typhoid, not more than fifteen grains a day being given. The kidneys are the best agents for the elimination of waste products. He gives abundant drinks, preferably lemonade made with wine, to favor diuresis.

In studying the influence of the treatment on mortality, he finds that the difference under the various methods is slight ; in 1889, under symptomatic treatment, there was a mortality in Paris hospitals of 11.33 per cent. ; by systematized treatment with cold baths, the mortality was 11.28 per cent. The lowest mortality for the year, 7.33 per cent., was obtained by the combined use of quinine and tepid baths.—*University Medical Magazine, March*, 1891.

ELECTRICITY IN GYNECOLOGY.—Apostoli (*Proceedings International Medical Congress*, 1890) states the following as his present views on the subject:

1. The principal value of the constant current is in its action in cases of fibroid tumors and endometritis, especially where pain and hemorrhage are constant symptoms. It not only arrests the growth of benignant tumors, but promotes the absorption of peri-uterine exudations, contraindicated in acute suppurative inflammation of the adnexa.

2. The galvanic current possesses a polar and an interpolar action, the latter being of a trophic and dynamic character, the former thermic and antiseptic.

3. The more powerful currents (above fifty milliamperes) influence the circulation through the development of heat. They are antiseptic and germicidal. They can be easily employed by the general profession. The stronger the current employed, the less the probability of a return of the symptoms.

4. Intra-uterine galvanization is preferable, because by it the maximum effect is obtained, the antiseptic action of the positive pole is secured, direct cauterization is effected, and there is less pain than in the intra-vaginal method.

5. In proper cases galvano-puncture by means of a fine gold needle, insulated to within one-fifth of an inch of its point, enables the operator to concentrate the action better and to produce a more powerful effect with weak currents.

6. As compared with the application of caustics and curetting, intra-uterine galvanization is far more harmless, as shown by the writer's experience, who, between July, 1882, and July, 1890, had practiced the latter 11,499 times with only three fatal results. One of these was a case of galvano-puncture for subperitoneal fibroma; another, the same for salpingo-oöphoritis; and in the other the same treatment was employed in an ovarian cyst which was mistaken for a fibroid.

In thirty instances pregnancy followed the use of intra-uterine applications.—*American Journal of Medical Sciences.*

FIRST ANNUAL REPORT OF THE NEW YORK PASTEUR INSTITUTE.—Eight hundred and twenty-eight persons, having been bitten by dogs or cats, came to be treated. These patients may be divided in two categories :

1st. For 643 of these persons it was demonstrated that the animals which attacked them were not mad. Consequently the patients were sent back after having had their wounds attended, during the proper length of time, when it was necessary.

2d. In 185 cases the anti-hydrophobic treatment was applied, hydrophobia of the animals which inflicted bites having been evidenced clinically, or by the inoculation in the laboratory, and in many cases by the death of some other persons or animals bitten by the same dogs. No death caused by hydrophobia has been reported among the persons inoculated.

Indigents have been treated free of charge.

The persons treated were : Eighty-one from New York, twenty-seven from New Jersey, sixteen from Massachusetts, eleven from Connecticut, nine from Illinois, five from Georgia, five from North Carolina, five from Pennsylvania, three from Maryland, three from Missouri, two from New Hampshire, two from Texas, two from Kentucky, two from Ohio, one from Maine, one from Arizona, one from Minnesota, one from Iowa, one from South Carolina, one from Nebraska, one from Rhode Island, one from Arkansas, one from Virginia, one from Louisiana, one from Indian Territory, one from Ontario, Canada.

---

DISEASES OF NORTHERN CALIFORNIA INDIANS.—Lieut. Charles E. Woodruff, M. D., U. S. A. (*Medical Record*, January 24), gives his experience and observations on certain diseases, particularly phthisis, among the Western Indians. It is not certainly known, he says, whether the disease prevailed before the advent of the white man. Nevertheless at the present time consumption is exceedingly prevalent and fatal among them. During a short residence among them of one year and a-half he has seen whole families wiped out of existence. It is among the half-breeds, white father and Indian mother, that the greatest mortality is found.

The changes wrought by the whites are certainly to some degree responsible for the increased prevalence. Formerly, while naked, he was inured to cold; would wade through mud and water with impunity, for in a few minutes his naked feet and legs would be dry again. Now, when clothed and shod, he does the same thing, but he has not learned to change his clothing, and will stand idly around until he dries. They crowd together in their rooms for warmth, and a consumptive among them has every facility for spreading the disease.

The children that are taken in white families and raised as servants, though originally possessed of vigorous health, after some years of civilization under the best conditions, fall into consumption and promptly die.

Syphilis and gonorrhœa are also exceedingly common. Syphilis and phthisis are probably responsible for the smallness of their families. Mortality of infants is large. The Indian seems to afford a natural culture ground for gonorrhœa. There is scarcely a squaw under sixty who has not had it, and the men accept it as a matter of course.

---

STRICTURE OF THE URETHRA—THE *University Medical Magazine* for March prints a valuable paper on Stricture of the Male Urethra, by Dr. J. William White, which concludes as follows:

1. Strictures of large calibre, that is, of more than 15 French, situated at or behind the bulbo-membranous urethra, are to be treated, almost without exception, by gradual dilatation.

2. Strictures of large calibre occupying the pendulous urethra are to be treated by gradual dilatation when very recent and soft, and by internal urethrotomy when of longer standing, distinctly fibrous in character or non-dilatable. It is to be remembered that the great majority of so-called strictures of large calibre of the pendulous urethra are merely points of physiological narrowing.

3. Strictures of the meatus and of the neighborhood of the fossa navicularis should be divided upon the floor of the urethra whenever it is evident that they are real pathological conditions producing definite symptoms, and not normal points of narrowing.

4. Strictures of small calibre (less than 15 French), situated in advance of the bulbo-membranous junction, unless seen very early and found to be unusually soft and dilatable, furnish the typical condition for internal urethrotomy, which should be done preferably with a dilating urethrotome and, invariably, with all possible antiseptic precautions.

5. Strictures of small calibre (less than 15 French), situated at, or deeper than, the bulbo-membranous junction, should be treated whenever possible by gradual dilatation. In a case of resilient, irritable or traumatic stricture in this region, or of stricture which, for any reason (as the occurrence of rigors), is non-dilatable, external perineal urethrotomy is the operation of choice.

6. Strictures of the deep urethra, permeable only to filiform bougies, should be treatd by gradual dilatation when possible, the filiform being left *in situ* for some time, and followed by the introduction of others, or used as a guide for a tunnelled catheter. If the stricture be not suitable for dilation, external perineal urethrotomy should be performed.

7. Impassable strictures of the deep urethra always require the performance of perineal section.

---

TREATMENT OF WHOOPING-COUGH.—The following treatment is used very largely by certain leading specialists in diseases of children in Paris. The treatment is divided into three periods. The patient should remain in one room or bed. The following prescription is given:

| | | |
|---|---|---|
| ℞. | Tincture of aconite, . . . . . | Of each 1 drachm. |
| | Tincture of belladonna, . . . | |
| | Camphorated tinct. opium, . | |

M. Sig.—Two to five drops once or twice a day, according to the age of the child.

If there is no febrile movement the amount of aconite may be much decreased, and if constipation is present the opium should not be used. In the second period, or when vomiting comes on, ipecac may be given in small amounts to allay gastric irritation, and in the third period, when convalescence is established, cod liver oil, tonics and Fowler's solution will be found of service.—*Medical News.*

---

TREATMENT OF CHOLERA INFANTUM.—In *Therapeutic Gazette* (Nov. 15, '90) Dr. L. G. Broughton, of Reidsville, N. C., recommends the following mixture in severe cases of cholera infantum, with profuse and watery stools :

℞. Salicylate of bismuth, . . . . 2 drachms.

Sulpho-carbolate of zinc, . . . 4 grains.

Chalk mixture, . . . . . . . 1 ounce.

Paregoric, } of each, . . . ½ ounce.

Water, }

M. Sig.—One drachm every two hours until bowels are controlled.

Then the following is given:

℞. Calomel, . . . . . . . . . . . . . 1 grain.
Sulpho-carbolate of sodium, . . 20 grains.
Saccharated pepsin, . . . . . . 19 grains.

Divide in ten powders, and give one every three hours. If the stomach is not irritable, sulpho-carbolate of zinc is substituted for the sodium salt in the last prescription.—*Gaillard's Medical Journal.*

---

LOCAL ANÆSTHESIA FOR SLIGHT OPERATIONS.—For operations upon small abscesses, opening fistulous tracts, or removing superficial growths, it is recommended that local anæsthesia be secured by following spray :

℞. Chloroform, . . . . . . . 10 parts.
Ether (sulphuric), . . . 15 parts.
Menthol, . . . . . . . . . 1 part.

M.

The anæsthesia thus obtained lasts from two to ten minutes.—*Medical News.*

THE INUNCTION METHOD IN SYPHILIS.—The plan of treatment employed by LELOIR, of Lille (*Uni. Med. Magazine*), is as follows: The initial lesion is treated with applications of a mercurial preparation. Constitutional treatment, which is withheld until secondary symptoms appear, consists of daily inunctions of from thirty to sixty grains of mercurial ointment, and the first course is continued for a period of from six to ten months. An interval of freedom from treatment for three weeks to two months is then allowed, and the inunctions are again instituted and kept up until the end of the second year. To prevent the accumulation of the drug, a diaphoretic or a laxative is occasionally given; and in the exceptional cases in which headache or bone pains are severe, iodide of potassium in combination with the bromide is prescribed. After the end of the second year, the course depends upon the severity of the case. If symptoms have been absent for a long period, the inunctions are made every three months for ten days, and then the iodide of potassium is exhibited for several weeks, in doses of from thirty to forty-five grains daily. After the third or fourth year, if there has been absence of symptoms for one year, the inunctions are made twice a year for ten days, and followed by a course of the iodide as before. This plan is continued if the patient is seen after the fourth year.

Leloir avoids the internal administration of mercury, on the ground that it may give rise to unfavorable symptoms, and employs it only when there is some reason why the inunctions cannot be practiced. Hypodermic injection of mercurial preparations he seldom resorts to, and then only in hospital patients.

---

INTRA-UTERINE TAMPON FOR POST-PARTUM HEMORRHAGE.—Auvard (*Archiv de Tocologie*, December, 1890) considers Duehrsen's plan of treating post-partum hemorrage by an intra-uterine tampon of iodoform gauze a safe and reliable treatment. He finds the mortality in sixty-seven cases about 6 per cent.

The method of applying the tampon is as follows: The anterior and posterior lips of the cervix are transfixed and drawn downward with tenacula, and a strip of iodoform gauze carried by means of dressing forceps to the fundus. The other hand is placed on the fundus through the abdominal wall, while the cavity of the uterus is being filled with the gauze. The tenacula are removed, and the end of the gauze is left at the vulvar opening. The tampon should be removed in from twelve to twenty-four hours. He considers two grades of post-partum hemorrhage—viz., bleeding of moderate severity, and hemorrhage—alarmingly profuse.

In the former variety the loss of blood may be due to uterine inertia, wounds of the vulva, vagina or cervix ; and the treatment of these milder cases should include, besides ligatures and sutures, antispetic injections of hot water, the administration of ergot and the application of the utero-vaginal tampon. When the loss of blood is alarming, uterine inertia is the cause. The bleeding should be controlled by compression and massage of the uterus through the abdominal wall, by the introduction of the hand into the uterus to remove its contents, followed by the utero-vaginal tampon.—*Univ. Med. Magazine.*

---

TREATMENT OF DIPHTHERIA.—Following is the London Hospital formula:

R. Ferri chloridi........... 6 drachms.
Potassii chloratis........40 grains.
Glycerini................ 4 drachms.
Aquæ q. s. ad........... 8 ounces.

Ft. Solutio. Sig. One-half to one teaspoonful every hour.—*Brit. Medical Journal.*

---

A MIXTURE FOR COLIC.—Dujardin-Beaumetz recommends the following mixture in the treatment of simple colic:

R. Strong chloroform water........4 ounces.
Decoction of orange flowers.......4 ounces.
Tincture of capsicum............2 drachms.

M. Sig. A dessertspoonful every 15 minutes until relieved.—*Medical News.*

### MEDICAL ITEMS.

IN the University of Pennsylvania the compulsory course for the medical degree has been lengthened to four years.

THE medical faculty of the University of Bonn has abandoned the use of both Koch's and Liebreich's "remedies" for tuberculosis.—*Medical Record.*

THE 38th annual meeting of the North Carolina State Medical Society will be held in Asheville May 26th, 27th and 28th. Subject for debate, "Appendicitis." Leader of debate, W. C. Galloway, M. D.

THE latest remedy for pulmonary consumption is that recently announced by Prof. Liebreich, of Berlin. It consists of Spanish fly in the form of the cantharidinate of potash subcutaneously. Surprisingly good results are obtained, particularly in tuberculosis of the larynx.

BLACK EYE.—There is nothing to compare with a tincture or a strong infusion of capsicum annuum, mixed with an equal bulk of mucilage of gum arabic, and with the addition of a few drops of glycerine. This should be painted all over the bruised surface with a camel's hair pencil, and allowed to dry, a second or third coat being applied as soon as the first is dry. If done as soon as the injury is inflicted this treatment will prevent the blackening of the bruised tissues. The same remedy has no equal in rheumatic, sore or stiff neck.—*Medical Times, Weekly Medical Review.*

A PLEA FOR CIRCUMCISION.—It is surely not needful to seek any recondite motive for the origin of the practice of circumcision. No one who has seen the superior cleanliness of a Hebrew penis can have avoided a very strong impression in favor of the removal of the fore-skin. It constitutes a harbor for filth, and is a constant source of irritation. It conduces to masturbation, and adds to the difficulties of sexual continence. It increases the risk of syphilis in early life, and cancer in the aged. I have never seen cancer of the penis in a Jew, and chancres are rare. —*Jonathan Hutchinson.*

---

J. P. Lippincott Company will, beginning with April, issue quarterly thereafter a work entitled "International Clinics." This work will comprise the best and most practical clinical lectures on medicine, surgery, gynæcology, pediatrics, dermatology, laryngology, ophthalmology and otology, delivered in the leading medical colleges of this country, Great Britain and Canada. These lecteres have been reported by competent medical stenographers and thoroughly revised by the professors and lecturers themselves. The object of the work is to furnish the busy practitioner and medical student with the best and most practical clinical instruction, in concise form. Each volume will consist of over 350 octavo pages, illustrated with photographic reproductions of important cases.

---

THE Illinois State Board of Health has recently refused longer to admit foreign physicians to practice in that State without undergoing the usual examination. This action was taken because: 1. The diplomas of medical schools and universities do not entitle the holders to practice in those countries. 2. As may be seen from p. viii of the "Report on Medical Education" of the Illinois State Board of Health for 1891, the Prussian Staats Examer Commission rejected in 1890 more than 40 per cent. of the graduates of the University of Berlin, more than 47 per cent. of the Breslau graduates, more than 31 per cent. of the Griefswald and Halle graduates, and in fact more than 29 per cent. of the university graduates that came before the commission. 3. Many of the rejected candidates come to this country. 4. Many such graduates, fearful of failing in the government examinations in their own countries, come to this country to enjoy a privilege denied them at home of practicing medicine simply on their diplomas. 5. The Illinois State Board of Health feels that it should not place upon diplomas a higher valuation than is given to them in the countries in which they are granted.—*Jour. Am. Med. Ass.*—*Weekly Med. Review.*

